# Risk management: Are there parallels between COVID19 and floods?

**DOI:** 10.1111/jfr3.12618

**Published:** 2020-05-13

**Authors:** Burrell E. Montz

As I write this, like millions of others around the world, I am sheltering in place hoping that we are able to get the coronavirus situation under control in the not‐too‐distant future. As more and more information (and misinformation) becomes available daily about COVID19, given my decades of experience with research on various aspects of flood hazards, it has been impossible for me to avoid thinking about flood risks and what we know and do not yet know about reducing vulnerabilities and losses and about how we communicate, understand, and manage risks. Clearly, the risks associated with COVID19 are very different than flood risks given the invisible nature of the former and the fact that everyone is vulnerable. Still, there are some parallels. Among them are how people perceive their risk and how those perceptions influence their decisions and behavior (including, in some cases, a false sense of security); the spatial and temporal patterns of risk and vulnerability, relating to both physical (biological in the case of coronavirus) and sociodemographic characteristics (in both cases the poor and elderly are at greater risk); and options and opportunities for managing or controlling the risk. COVID19 is a risk with which, until recent months, we have very little experience, and we have so very much to learn. With flooding, we have a great deal of experience, yet we still have a great deal to learn.

It is through research and putting the results of that research into action that we can reduce risks. At the same time, despite the thousands (I am guessing!) of research projects and publications on flood risks and extensive public and private efforts in reducing that risk, losses continue to increase. We can point to some obvious reasons why. Populations are increasing, while risk‐free or lower‐risk locations are not. Flood risk may not be seen to be sufficiently high to merit attention given other salient issues or problems that we are facing. The technology to reduce losses is increasing, but so too are the costs, making it difficult for implementation in many places. These are but a few explanations. We also know that flood risks vary from place to place and time to time, so there is no single solution to address any of the reasons I have just listed. We have clearly been making progress as we have seen research translated into practice. For instance, advances in geographic information science (GIS) and remote sensing have led to the development of more accurate maps of flood risk areas for insurance and land use management purposes. Similarly, surveys of residents' perceptions of risk have fostered changes in warnings to spur appropriate protective action. Again, these are but a few. We need to continue to ask important questions and undertake the research to answer them—addressing different components of flood risk in different locations with different political, economic, and socioeconomic contexts. The papers in this issue do just that: address different components of the flood risk problem in different locations, with an eye to reducing vulnerabilities and thus losses.

Put simply, risk management is a process that might be summed up as having three components to it: identifying exposure, identifying and evaluating options, and comparing and choosing options. These are broad categories that require consideration by a wide range of disciplines, addressing very diverse elements. For instance, identifying exposure involves hydrological, engineering, spatial, and sociodemographic analyses documenting what and who is exposed and to what extent. A few examples from this issue illustrate these points. From a hydrological perspective, Alipour, Ahmadalipour, and Moradkhani (2020) address exposure through the use of a 20‐year database encompassing 14,000 flash flood events to document spatial and seasonal patterns in the southeastern United States, while Chen, Giese, and Chen (2020) analyze a 30+‐year database of flood occurrence in mainland Southeast Asia to understand temporal changes in flood occurrence. Finer‐scale means of identifying exposure have often been undertaken using GIS, as noted above. However, the associated costs may be prohibitive in some places. Thus, lower‐cost methods have been explored, an example being a case study in Haiti using open‐source and field data, among other sources, to develop economic and social risk maps (Glas, De Maeyer, Merisier, & Deruyter, 2020).

So far, I have emphasized the *where* of exposure from which the *who* and *what* can be identified. However, there is also the issue of interrelationships among the where, who, and what. Locations at low risk of floods can experience significant impacts if the infrastructure on which they depend is compromised, as illustrated by Fekete (2020), who addresses the cascading effects on critical infrastructure, and Hathout, Peyras, Carvajal, Diab, and Vuillet (2020), who present a novel way to evaluate levee failure probabilities. The discussion, above all, has relevance beyond identifying exposure as it feeds into identifying and evaluating options. Available options may center on developing options to move people out of harm's way, a topic considered here by Wang, Shi, and Zhou (2020), or evaluating the impacts and viability of various flood control options such as channelizing (Juan, Gori, & Sebastian, 2020).

It is from the results of studies such as those in this issue (and many others) that options to manage risk can be compared and choices can be made by policymakers working within their political and economic contexts. We have seen much the same with COVID19: analyses of exposure spatially, temporally, and demographically; cascading impacts; and policymakers weighing options to manage the risk, often basing the choice of options on the results of models of the outcomes of various interventions. The results of work on flood risk management do not have direct application to COVID19, but there are certainly parallels in how the risk is addressed. And we know from the successes in managing flood risk that it is through individual studies of the various components of risk that we make strides forward.

## References

[jfr312618-bib-0001] Alipour, A. , Ahmadalipour, A. , & Moradkhani, H. (2020). Assessing flash flood hazard and damages in the southeast United States. Journal of Flood Risk Management, 13(2), e12605.

[jfr312618-bib-0002] Chen, A. , Giese, M. , & Chen, D. (2020). Flood impact on Mainland Southeast Asia between 1985 and 2018—The role of tropical cyclones. Journal of Flood Risk Management, 13(2), e12598.

[jfr312618-bib-0003] Fekete, A. (2020). Critical infrastructure cascading effects. Disaster resilience assessment for floods affecting city of Cologne and Rhein‐Erft‐Kreis. Journal of Flood Risk Management, 13(2), e312600.

[jfr312618-bib-0004] Glas, H. , De Maeyer, P. , Merisier, S. , & Deruyter, G. (2020). Development of a low‐cost methodology for data acquisition and flood risk assessment in the floodplain of the river Moustiques in Haiti. Journal of Flood Risk Management, 13(2), e12608.

[jfr312618-bib-0005] Hathout, M. , Peyras, L. , Carvajal, C. , Diab, Y. , & Vuillet, M. (2020). Development of an approach to evaluate the failure probabilities of river levees based on expert judgement: Application to a case study. Journal of Flood Risk Management, 13(2), e12603.

[jfr312618-bib-0006] Juan, A. , Gori, A. , & Sebastian, A. (2020). Comparing floodplain evolution in channelized and unchannelized urban watersheds in Houston, Texas. Journal of Flood Risk Management, 13(2), e12604.

[jfr312618-bib-0007] Wang, N. , Shi, G. , & Zhou, X. (2020). To move or not to move: How farmers now living in flood storage areas of China decide whether to move out or to stay put. Journal of Flood Risk Management, 13(2), e312609.

